# Stroke‐Homing Peptide‐DNase1 Alleviates Intestinal Ischemia Reperfusion Injury by Selectively Degrading Neutrophil Extracellular Traps

**DOI:** 10.1111/cpr.70010

**Published:** 2025-02-25

**Authors:** Tingting Liu, Xinrong Lv, Qingshan Xu, Xiuting Qi, Shenghui Qiu, Yaqi Luan, Na Shen, Jing Cheng, Lan Jin, Tian Tian, Wentao Liu, Lai Jin, Zhongzhi Jia

**Affiliations:** ^1^ Graduate College of Dalian Medical University Dalian Liaoning China; ^2^ Department of Interventional and Vascular Surgery The Third Affiliated Hospital of Nanjing Medical University (Changzhou Second People's Hospital) Jiangsu China; ^3^ Department of Neurobiology, School of Basic Medical Sciences Nanjing Medical University Nanjing Jiangsu China; ^4^ School of Basic Medical Science Nanjing Medical University Nanjing Jiangsu China; ^5^ Department of Nephrology Shandong Provincial Hospital Affiliated to Shandong First Medical University Jinan Shandong China; ^6^ Department of Gastroenterology Shanghai General Hospital of Nanjing Medical University Shanghai China; ^7^ Department of Gastroenterology Lianyungang Municipal Oriental Hospital Lianyungang China; ^8^ Innovation Center of Suzhou Nanjing Medical University Suzhou Jiangsu China; ^9^ National Center of Technology Innovation for Biopharmaceuticals Suzhou Jiangsu China

**Keywords:** ceftriaxone sodium, intestinal ischemia reperfusion injury, microcirculatory disturbance, neutrophil extracellular traps, stroke‐homing peptide–DNase1

## Abstract

Neutrophil extracellular traps (NETs) act as a vital first line of defence against tissue damage and pathogens, playing a significant role in improving diseases such as intestinal ischemia reperfusion injury (IRI). However, we observed that after intestinal injury, intestinal bacteria and lipopolysaccharides (LPS) can enter the circulatory system, leading to a significant secondary increase in NETs production and the subsequent activation of a coagulation cascade. This phenomenon contributes to a pathological process known as the ‘second strike’ of NETs, which exaggerates intestinal damage and microcirculation disturbance. Selectively mitigating the detrimental effects associated with this second strike presents a promising therapeutic strategy. We developed an innovative conjugate of stroke‐homing peptide (SHp) and DNase1 (SHp‐DNase1) to enhance the stability of DNase in the bloodstream while selectively targeting NETs in thromboembolic events. The effects of SHp‐DNase1 on blood flow, ischemia, and vascular leakage were evaluated in a mouse model using laser Doppler flowmetry and an in vivo imaging system. Levels of LPS and NETs were elevated in patients with IRI. Similarly, the expression of NETs and LPS was upregulated in mice with intestinal IRI. In vivo imaging revealed disturbances in intestinal microcirculation, accompanied by intestinal leakage, which were effectively reversed by the administration of SHp‐DNase1. Almost all of the SHp‐DNase1 localised to the gastrointestinal tract, demonstrating the effective targeting of DNase1 to the site of intestinal injury via SHp guidance. Furthermore, the combination of SHp‐DNase1 and CRO significantly reduced the expression of ischemia‐inducible factors, leading to a marked decrease in mortality in the mouse model. These findings suggest that intestinal LPS leakage correlated with NETs exacerbation plays a critical role in IRI. The combination of SHp‐DNase1 and CRO is an effective treatment strategy by simultaneously controlling inflammation and addressing microcirculatory disorders induced by NETs in the therapy of IRI.

## Graphical abstract

SHp‐DNase1 combined with CRO inhibits NETs to alleviate intestinal IRI

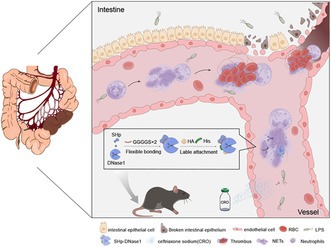

## 1 Introduction

Mesenteric vascular ischemia is a life‐threatening condition that requires immediate management. Restoring perfusion as quickly as possible is the most important salvage measure to treat bowel ischemia and save bowel tissue. However, once the blood supply is restored, a more serious complication known as intestinal ischemia reperfusion injury (IRI) can occur [1], particularly in patients with acute and critical conditions such as mesenteric artery occlusive disease or haemorrhagic shock and in those who have undergone intestinal transplant or cardiopulmonary bypass (CPB) [2, 3]. In cases of IRI, reperfusion after intestinal ischemia leads to localised damage to intestinal tissues, as well as intestinal flora disruption and bacterial translocation, and endotoxin release due to damage to the intestinal epithelial barrier [4, 5]. This in turn can lead to systemic inflammatory response syndrome, multiorgan dysfunction, and even death [6]. Indeed, the mortality rate in patients with intestinal IRI may be as high as 60% to 80% [7]. A thorough understanding of the mechanism underlying intestinal IRI is therefore needed.

In the setting of injury or infection, neutrophils are the first immune cells to be recruited to the site [8]. Activated neutrophils are stimulated to produce and release neutrophil extracellular traps (NETs), which mainly consist of DNA fibres wrapped in histones, myeloperoxidase (MPO), and neutrophil elastase (NE), amplifying their antimicrobial effectiveness [9]. However, NETs are a double‐edged sword [10]. In pathological processes such as IRI, increased levels of plasma lipopolysaccharides (LPS) and pathogens resulting from intestinal leakage can lead to excessive production and inadequate clearance of NETs, which are often accompanied by tissue damage. We refer to this clinical phenomenon as the ‘second strike’ initiated by NETs. Excessive NETs create a physical scaffold for platelets and fibrin, facilitating thrombus formation and the aggregation of reticulated red blood cells, which can obstruct capillaries [11]. Moreover, recent research has identified elevated levels of NETs as the key factor that triggers the phosphorylation of Fundc1 at Tyr18 in intestinal endothelial cells, resulting in endothelial ferroptosis and microcirculatory dysfunction [12]. This damage to microvascular structures can compromise the intestinal barrier, further exacerbating the injury processes associated with intestinal IRI and creating a vicious cycle [13]. Therefore, targeting NETs during ischemia reperfusion may represent a promising approach for the treatment of intestinal IRI. Physiologically, NETs are degraded by serum DNase such as DNase1, which is the dominant endonuclease in plasma [14]. A study in an intestinal IRI rat model demonstrated that DNase1 treatment reduced extracellular DNA in the form of NETs associated with organ damage [15]. Additionally, DNase1 has been shown to attenuate the inflammatory response, upregulate the expression of intestinal tight junction proteins, and restore intestinal barrier integrity [16]. However, low serum stability and rapid inactivation by environmental stimuli are limiting factors for the clinical application of DNase1 [14]. Thus, targeted delivery strategies that rapidly and accurately deliver DNase1 to sites at which NETs cause microcirculatory impairment are urgently needed.

In our recent study, we found that a stroke‐homing peptide (SHp; CLEVSRKNC) [17] coupled with DNase1 would specifically anchor at the site of microcirculatory ischemia in the lower limbs, effectively alleviating mechanical hyperalgesia [18]. Based on these findings, we aimed to develop a delivery system consisting of a recombinant protein of SHp with human DNase1 (SHp‐DNase1). We then tested the ability of this system, with or without the addition of the cephalosporin antibiotic ceftriaxone sodium (CRO), to target the degradation in pathological NETs specifically at the thrombus site, thereby ameliorating intestinal microcirculatory disturbances in the context of intestinal IRI.

## 2 Materials and Methods

### 2.1 Patients and Clinical Data

This study was approved by the Institutional Review Board of The Third Affiliated Hospital of Nanjing Medical University (Changzhou Second People's Hospital), with registration number [2024]KY206‐01, and all methods were performed in accordance with relevant guidelines and regulations. A total of 7 patients who underwent CPB procedures during cardiac surgery were included in the study, as CPB may lead to intestinal ischemia and injury due to reduced blood supply and oxygen delivery [19, 20]. The baseline characteristics of the patients with CPB are summarised in Table S1. The median (range) of the patients was 67 (41–77) years, and 71.42% of patients were female. Patients were excluded from this analysis if they were aged < 18 years, if they had chronic kidney disease, or if they had chronic gastrointestinal disease, had undergone previous gastrointestinal surgery, or had confirmed or suspected intestinal ischemia/necrosis [21].

Blood samples were collected before the surgery and at 8, 24, and 72 h after the surgery. The samples were centrifuged at 3000 × g for 10 min at 4°C to obtain serum samples, which were then stored at −80°C.

### 2.2 Enzyme‐Linked Immunosorbent Assay (ELISA)

Serum intestinal fatty acid binding protein (i‐FABP) and LPS levels are objective predictors of postoperative prognosis in patients undergoing cardiac surgery; high serum levels of these biomarkers indicate the presence of intestinal injury [21, 22]. ELISA was used to assess the levels of these markers in study patients. Briefly, the blood samples were centrifuged at 3000 × g for 10 min, and the levels of cell‐free (cf)‐DNA (Thermo Fisher Scientific, Waltham, Massachusetts, USA), LPS (Boshen, Nanjing, China), citrullinated histone H3 (H3Cit) (Boshen, Nanjing, China), and i‐FABP (Bioswamp, Wuhan, China) were evaluated based on the ELISA kit instructions.

### 2.3 Preparation of SHp‐DNase1

For targeted delivery of DNase1, SHp was designed to fuse with DNase1 with a 2× GGGGS linker sequence. The designed SHp‐linker‐DNase1‐HA‐His6 sequence flanked with the NheI site was synthesised and cloned into pCSCW‐IG, a lentivirus gene transfer plasmid that expresses GFP separately by an internal ribosome entry site (IRES) element under the control of the cytomegalovirus (CMV) promoter (pCSCW‐CMV‐SHp‐linker‐DNase1‐HA‐His6‐IRES‐GFP). The lentivirus was then generated with the designed plasmids. HEK293T cells were stably transduced with the packaged lentivirus to express SHp‐DNase1. The cells were cultured for 48 h, and the conditional medium was then harvested. After removal of cells and debris by centrifugation at 10,000 g for 20 min at 4°C, the supernatant was concentrated using a 10‐kD ultrafiltration tube (Millipore). SHp‐DNase1 was purified using a His‐tag Protein Purification Kit (Beyotime, Shanghai, China) according to the manufacturer's instructions. In addition, SHp was synthesised with Cy5.5 fluorophore conjugation, and the labelled peptide was used to track the site of SHp accumulation in vivo.

### 2.4 Animals and Intestinal IRI Model

Wild‐type (WT) male C57BL/6J mice weighing 20 to 25 g and aged 6 to 8 weeks were purchased from Charles River Laboratories (Zhejiang, China), and male peptidyl arginine deiminase 4 knockout (*Pad4* ^*−/−*^) mice on the C57BL/6J background were purchased from The Jackson Laboratory. PAD4 is a key enzyme involved in the citrullination of histone proteins to form NETs [23]. To further validate the critical role of NETs in the microcirculatory disturbances caused by intestinal IRI, we therefore used *Pad4* ^*−/−*^ mice. All animals had free access to food and water and were acclimated to a controlled temperature (22°C ± 2°C) and photoperiod (12‐h light/12‐h dark cycle) for 1 week.

All mice were fasted for 12 h and given free access to water before the experimental procedure. A preoperative intraperitoneal injection of 1.2% Avertin (240 mg/kg) was used for anaesthesia. The mice were then placed in the supine position on an electric blanket and kept at a constant temperature. The abdominal hair was shaved, and the area was sterilised and prepared for surgery. A 1‐cm midline abdominal incision was made, and the small intestine was removed with a saline‐moistened swab to expose the superior mesenteric artery. The superior mesenteric artery was then clamped with a nontraumatic vascular clip for 40 min. Saline drops were applied to keep the bowel moist. The arterial clip was then removed to start reperfusion, and the abdominal wound was closed. Mice were checked at least every 30 min, and their respiration and heart rate were closely monitored. After reperfusion, the mice were deeply anaesthetised via inhalation of 5% isoflurane. Blood samples and a 5‐cm segment of small intestine at the end of the ileum (ileocecal valve) were collected at 2, 8, 24, and 72 h after reperfusion for further analysis [24].

Animals were randomly assigned to one of the following groups (*n* = 12–15): WT mice who underwent a sham procedure (superior mesenteric artery not clamped); WT mice with intestinal IRI; *Pad4* ^*−/−*^ mice with intestinal IRI; WT mice with intestinal IRI treated with CRO; WT mice with intestinal IRI treated with DNase1; WT mice with intestinal IRI treated with SHp‐DNase1; and WT mice with intestinal IRI treated with SHp‐DNase1 and CRO. In SHp‐DNase1–treated or DNase1‐treated mice, SHp‐DNase1 or DNase1 (200 μg/kg) was injected through the tail vein before reperfusion and again 8 h after reperfusion. Mice treated with CRO were injected intraperitoneally with CRO (200 mg/kg) 30 min before modelling.

### 2.5 Haematoxylin and Eosin (H&E) Staining

Fresh small samples of intestinal tissues from the mouse model were immediately fixed in 4% paraformaldehyde after being gently rinsed in saline. After undergoing dehydration through successive alcohol gradients, the segments were embedded in paraffin blocks and divided into 5‐μm thick sections. H&E staining was then performed [25]. Two independent pathologists, unaware of the experimental grouping, employed the Chiu scoring system to evaluate the intestinal mucosal damage in these sections [26]. A score of 0 was used to represent normal mucosa and glands; 1, damage to the apical part of the villi and widening of the subepithelial space; 2, further widening of the subepithelial space with detachment of the lamina propria; 3, epithelial detachment of the villi on both sides of the lamina propria in clumps; 4, the presence of lamina propria structures alone; and 5, lysis of the lamina propria of the mucosa with haemorrhage and ulceration.

### 2.6 Measurement of Blood Flow in the Intestines and Lower Limbs

Although intestinal damage may be gradually recovered after reperfusion, microcirculation disorders can still exist, causing a series of sequelae such as severe sepsis, septic shock, and even multiorgan dysfunction [12]. Thus, monitoring and protection of the microcirculation after intestinal ischaemia reperfusion have received increasing attention [27, 28]. For this study, laser Doppler flowmetry was used to measure blood flow in the intestine and limbs of mice. Specifically, a low‐power laser beam was directed through an optical scanner into the exposed small intestine and lower limbs of mice after the abdomen had been opened. Colour‐coded images indicating relative perfusion were displayed on a computer monitor. The Moor FLPIR V40 program (Gene & I Scientific. Ltd) was used to record and analyse the blood flow values.

### 2.7 In Vivo Imaging System (IVIS) Spectrum Imaging

Mice were injected with SHp‐Cy5.5 (20 μg/kg) in the tail vein 2 h before imaging (at 6 h of reperfusion) or treated with fluorescein isothiocyanate (FITC) (Sigma, 600 mg/kg) by gavage 4 h before imaging (at 4 h of reperfusion). Images of ischemic or leaky sites in mice were captured by the IVIS (PerkinElmer).

### 2.8 Immunofluorescence Assay

Intestinal tissues from the study mice were taken and fixed in 4% paraformaldehyde solution. Embedded blocks were sectioned at a thickness of 5 μm. The slides were sealed with 10% donkey serum (017–000‐121, Jackson ImmunoResearch) for 1 h and were then incubated overnight at 4°C with primary antibodies, including H3Cit (1:400; Abcam, Cambridge, UK), MPO (1:300; Abmart, Shanghai, China), and TF (1:300; Santa Cruz Biotechnology, Oslo, Norway). The samples were then washed 3 times with PBS. Alexa Fluor 488‐conjugated donkey anti‐rabbit or Alexa Fluor 647‐conjugated donkey anti‐mouse was added, and the samples were incubated for 2 h at room temperature. The samples were then washed and sealed, and morphological details of immunofluorescence staining were observed under a laser confocal microscope (Zeiss LSM880 with Airyscan). Investigators who were blinded to the test groups analysed the findings using ImageJ software.

### 2.9 Immunohistochemistry Analysis

Paraffin sections were heated in a microwave oven for 20 min, deparaffinised in xylene, and rehydrated through a gradient series of ethanol solutions. Endogenous peroxidase activity was blocked by incubation with anhydrous methanol solution of 0.3% hydrogen peroxide for 15 min at room temperature. Sections were then incubated with primary antibody overnight at 4°C. Zonula occludens (ZO)‐1 (1:400; Proteintech). The tissue sections were then washed, and the sections were incubated with secondary antibody for 1 h at room temperature. Staining was shown by DAB reaction. The tissues were counterstained with haematoxylin. Immunohistochemistry results were observed under a laser confocal microscope (Zeiss LSM880 with Airyscan), and analysis was performed using Image J software.

### 2.10 Western Blot Analysis

Proteins were extracted from intestinal tissue using lysis buffer (Beyotime). The lysis products were separated via sodium dodecyl sulfate‐PAGE and were then transferred to polyvinylidene fluoride membranes. The membranes were sealed with 5% skimmed milk (Beyotime) for 2 h at room temperature, and primary antibodies were then added and incubated overnight at 4°C. The primary antibodies were H3Cit (1:1000; Abcam), tissue factor (TF) (1:1000; Santa Cruz Biotechnology), hypoxia‐inducible factor (HIF)‐1α (1:1000; Cell Signalling Technology), MPO (1:1000; Abmart), matrix metalloproteinase (MMP)‐9 (1:1000; Proteintech), occludin (1:1000; Proteintech), and β‐actin (1:5000; Proteintech). After incubation with the secondary antibody for 2 h at room temperature, data were acquired using a molecular imager (ChemiDoc, Bio‐Rad), and the findings were analysed using Image J software.

### 2.11 Statistical Analysis

Statistical analyses were performed using GraphPad Prism. Unpaired *t*‐tests and one‐way ANOVA were used to determine quantitative statistical significance. All results were expressed as mean ± SD, and *p* < 0.05 was used to indicate statistical significance.

## 3 Results

### 3.1 Intestinal IRI Triggered NETs Formation

**FIGURE 1:**
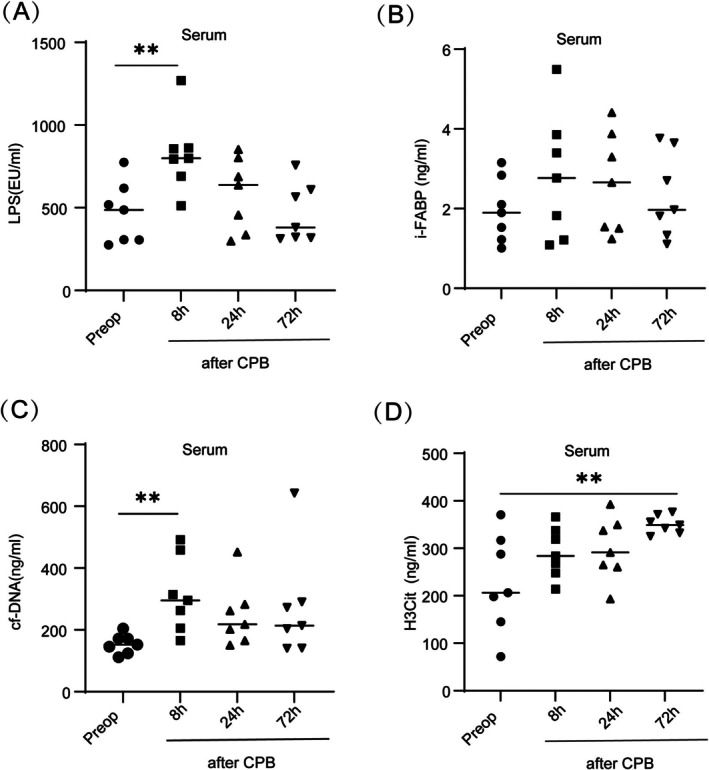
Neutrophil extracellular traps (NETs) in patients with intestinal ischemia reperfusion injury (IRI). (A–D) Levels of lipopolysaccharide (LPS), intestinal fatty acid binding protein (i‐FABP), cell‐free (cf)‐DNA, and citrullinated histone H3 (H3Cit) in the serum of patients who experienced intestinal IRI after undergoing cardiopulmonary bypass (CPB) (*n* = 7). Levels were obtained via ELISA before CPB (Preop) and at 8, 24 and 72 h after CPB. **p < 0.01 (*t*‐test).

CPB cardiac surgery is associated with intestinal barrier dysfunction. Intestinal barrier dysfunction can be assessed non‐invasively by LPS and i‐FABP serum levels [22]. In the 7 study patients who underwent CPB and experienced intestinal IRI, LPS expression was significantly elevated after reperfusion, and the level of i‐FABP also showed an upward trend (Figure 1A,B). Cf‐DNA and H3Cit levels showed a similar dynamic peak change (Figure 1C,D). These findings suggest that intestinal IRI stimulated the release of NETs and that intestinal LPS may be the main stimulus for the generation of NETs.

**FIGURE 2:**
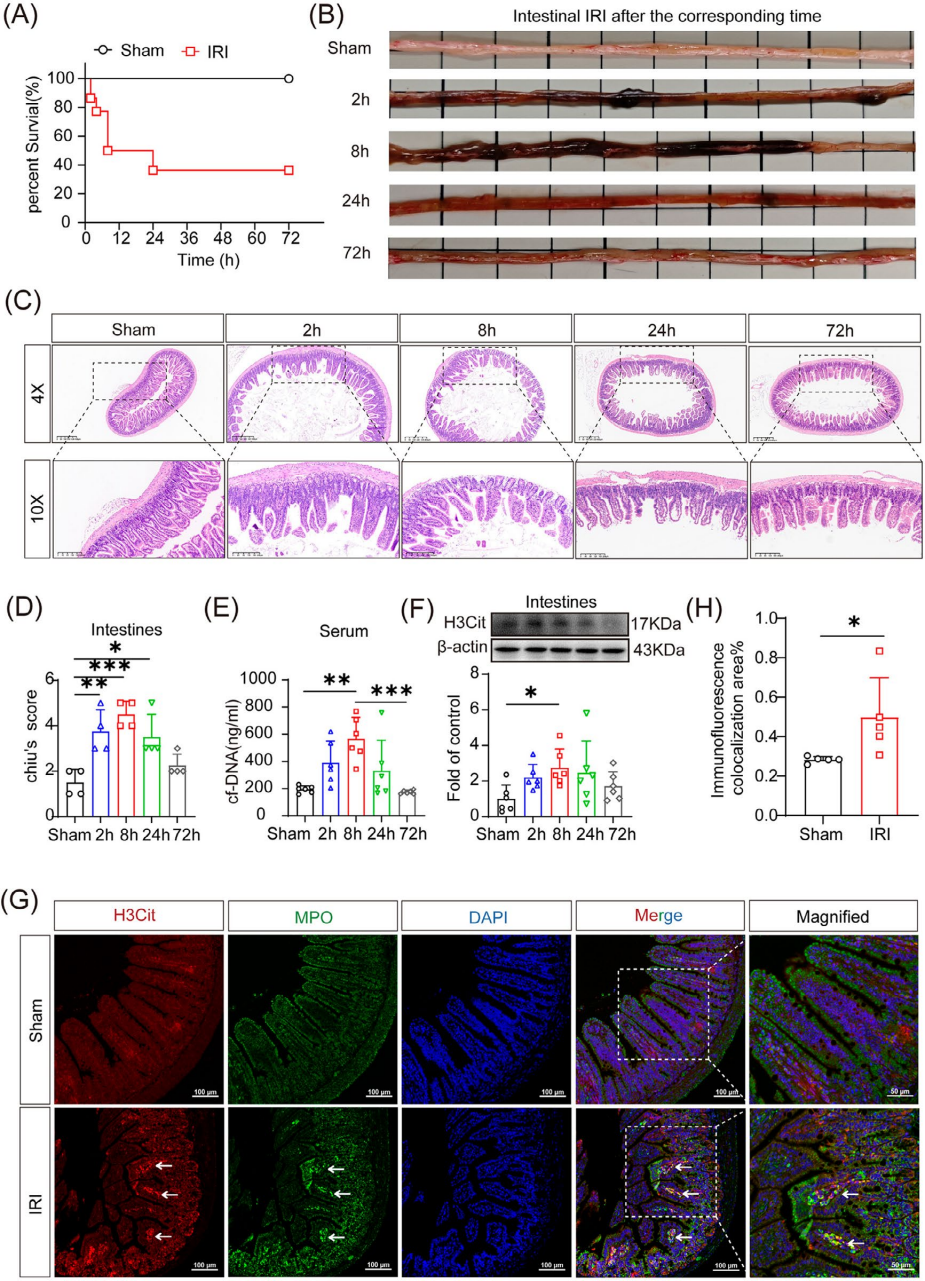
Intestinal ischemia reperfusion injury (IRI)‐induced neutrophil extracellular trap formation. (A) The survival rate was significantly lower in mice with intestinal IRI (*n* = 22, Sham vs. IRI *p* = 0.0048). (B) Macroscopic view of the intestine at different time points after reperfusion. (C) Representative images of H&E staining at 2, 8, 24, and 72 h after reperfusion. 4× scale bar = 400 μm; 10× scale bar = 200 μm. (D) Intestinal Chiu's score (*n* = 4). (E) Detection of cell‐free (cf)‐DNA level in serum by ELISA (*n* = 6). (F) Detection of citrullinated histone H3 (H3Cit) expression in intestine by Western blot (*n* = 6). (G) Typical confocal immunofluorescence microscopy image of intestine with myeloperoxidase (MPO) (green), H3Cit (red), and DAPI (blue) staining. The arrows point to the co‐localization of MPO and H3Cit. 10× scale bar = 100 μm; magnified scale = 50 μm (*n* = 5). (H) H3Cit co‐localised area with MPO as percentage of total fluorescence area. *p < 0.05, **p < 0.01, and ***p < 0.001 versus sham group (one‐way ANOVA [D–F] or *t*‐test [H]).

In the mouse model, more than 60% of mice with IRI died within 24 h of reperfusion (Figure 2A). Compared with the sham group, the intestines from these IRI mice showed obvious redness, swelling, haemorrhage, and necrosis at 2 and 8 h after reperfusion, with gradual recovery seen after 24 h (Figure 2B). The epithelium of the villi was apically broken at 2 h after reperfusion. At 8 h, the villi were almost completely detached, the lamina propria was dissolved, and the subepithelial gap was widened. At 24 and 72 h, the villi had recovered gradually (Figure 2C). The pathological scores for intestinal mucosal damage were significantly higher in the IRI group than in the sham group (Figure 2D). The serum level of cf‐DNA peaked at 8 h after reperfusion and then gradually declined (Figure 2E), and both western blotting and immunofluorescence analyses showed elevated expression of H3Cit in the intestine at 8 h after modelling (Figure 2F–H). Co‐localization of H3Cit and MPO further proved the abundance of NETs in the intestine. These results suggest that intestinal IRI led to a lethal injury, which was accompanied by the overproduction of NETs.

### 3.2 Intestinal IRI Induced Intestinal Microcirculation Disorders

**FIGURE 3:**
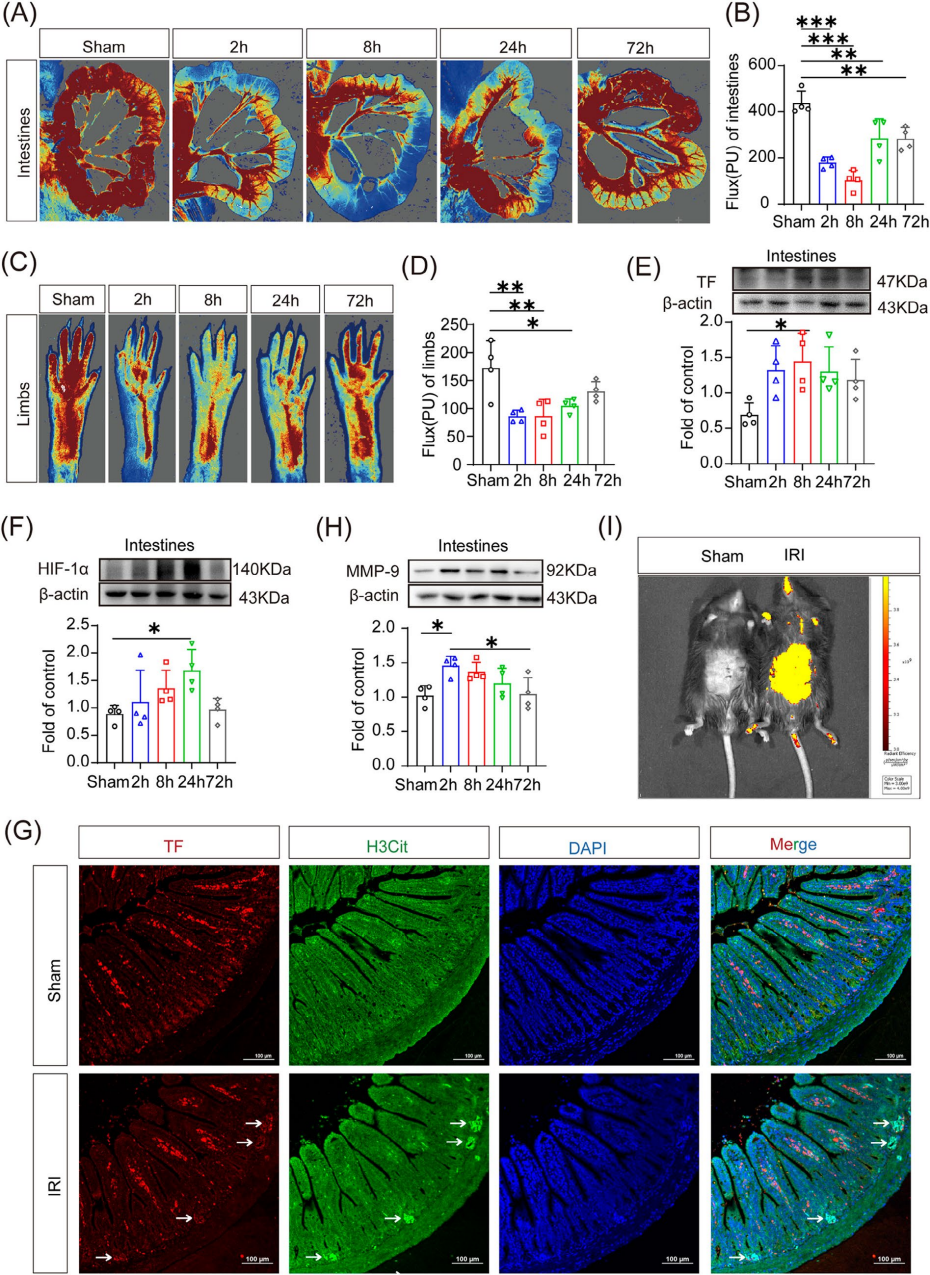
Intestinal ischemia reperfusion injury (IRI)‐induced intestinal microcirculation disorders. (A–D) Detection of intestinal (A, B) and extremity (C, D) blood flow using laser Doppler flowmetry (*n* = 4). (E, F) Detection of tissue factor (TF) (E) and hypoxia‐inducible factor (HIF)‐1α (F) expression by Western blot (*n* = 4). (G) Immunofluorescence showing the co‐localization of TF (red) and citrullinated histone H3 (H3Cit) (green). Arrows point to sites of co‐localization. Scale bar = 100 μm (*n* = 3). (H) Detection of matrix metalloproteinase (MMP)‐9 expression by Western blot (*n* = 4). (I) At 4 h after fluorescein isothiocyanate gavage (8 h after reperfusion), intestinal leakage was monitored using the In Vivo Imaging System. *p < 0.05, **p < 0.01, and ***p < 0.001 versus sham group (one‐way ANOVA).

Multiple activation of NETs promotes thrombus formation. After a period of reperfusion, there was a gradual decrease in intestinal blood flow in the WT mice with IRI, with significant microcirculatory disturbances (Figure 3A,B). The degree of microcirculatory disturbances was consistent with the level of NETs (Figure 2E,F). The blood flow in the lower limbs was also affected (Figure 3C,D), suggesting the importance of microcirculatory recovery. The expression of TF and HIF‐1α was markedly up‐regulated in the intestine (Figure 3E,F). Immunofluorescence staining of the intestinal tissues indicated a pronounced increase in TF level at 8 h after reperfusion, and TF was found to be partially co‐localised with H3Cit (Figure 3G), suggesting the involvement of NETs in microthrombi formation. The expression of intestinal MMP‐9 was also elevated in the modelling group of mice (Figure 3H). In addition, IVIS Spectrum imaging demonstrated evidence of severe intestinal leakage in the modelling group of mice (Figure 3I). These findings suggest that microthrombus contributes to microcirculatory disturbances after intestinal IRI, resulting in intestinal leakage and subsequent enterobacteria translocation, and that this microthrombus may be due to elevated NETs levels.

### 3.3 CRO Alleviated NETs‐Associated Microcirculatory Disturbances by Reducing LPS

**FIGURE 4:**
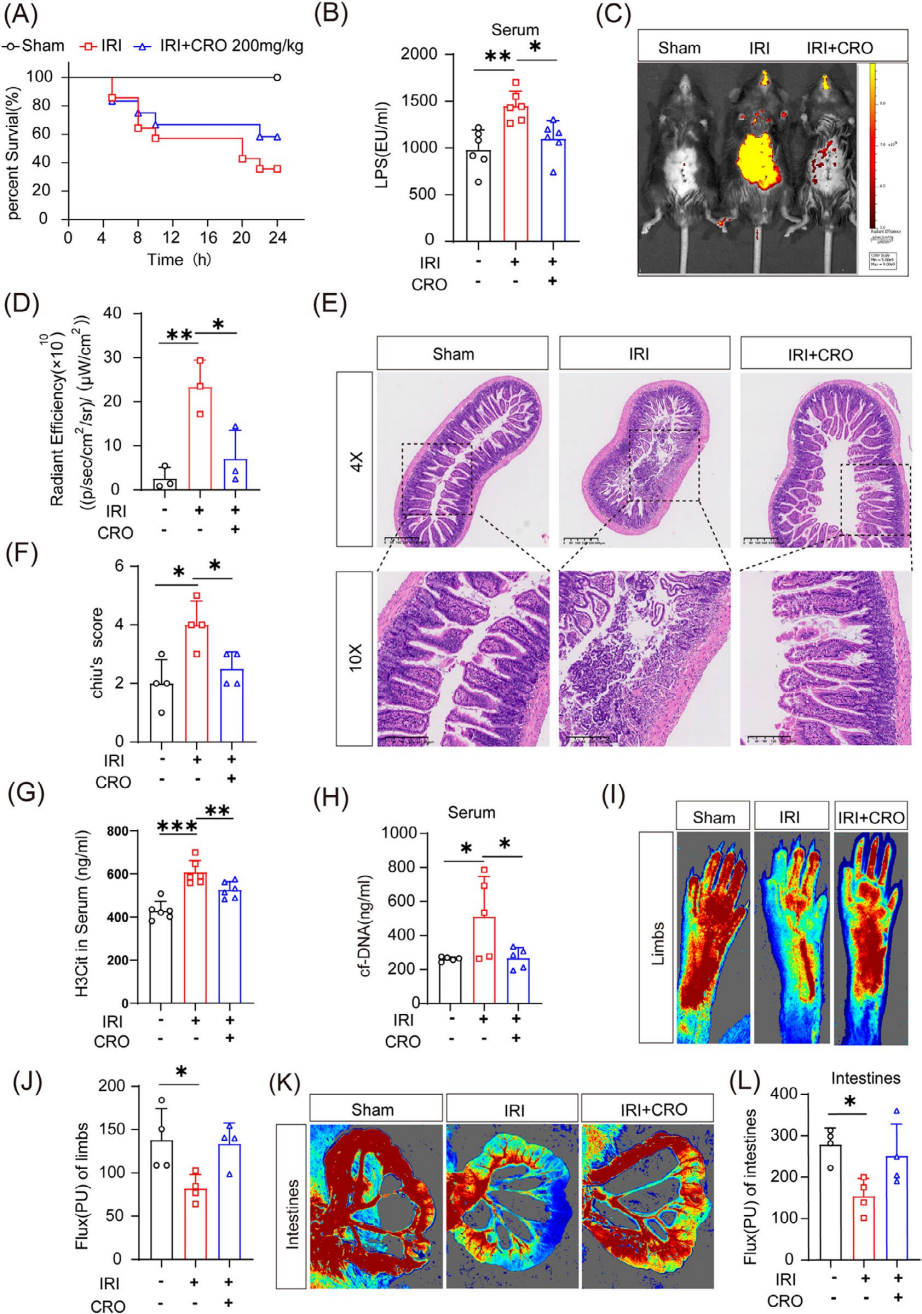
Gut‐derived lipopolysaccharide (LPS) was critical for intestinal ischemia reperfusion injury (IRI)‐induced neutrophil extracellular traps (NETs) formation. (A) Survival rate of mice within 24 h of ceftriaxone sodium (CRO) treatment (*n* = 12 in the sham group and IRI + CRO intervention group, *n* = 14 in the IRI group, Sham versus IRI *p* = 0.0156). (B) ELISA for serum LPS levels in mice after 8 h of reperfusion (*n* = 6). (C, D) At 8 h after reperfusion, intestinal leakage in mice was monitored using the In Vivo Imaging System (*n* = 3). (E) H&E staining of intestinal tissue (*n* = 4). 4× scale bar = 400 μm; 10× scale bar = 200 μm. (F) Chiu's score for intestinal injury (*n* = 4). (G) ELISA for the expression of citrullinated histone H3 (H3Cit) markers in NETs (*n* = 6). (H) ELISA for the expression of cell‐free (cf)‐DNA markers in NETs (*n* = 5). (I–L) Blood flow in intestines and limbs based on laser Doppler flowmetry (*n* = 4). *p < 0.05, **p < 0.01, and ***p < 0.001vs sham group (one‐way ANOVA).

Disruption of the intestinal mucosal barrier leads to a large number of bacteria and endotoxins in the intestinal lumen entering the blood circulation. Within 24 h of reperfusion, CRO was found to reduce mortality to a certain extent (Figure 4A). Serum LPS levels also decreased after reperfusion in mice treated with CRO (Figure 4B). IVIS Spectrum imaging demonstrated that FITC in the gastrointestinal tract of mice with IRI diffused into the abdominal cavity, and CRO treatment reversed this occurrence (Figure 4C,D). This suggests that the intestinal mucosal barrier was disrupted by intestinal microthrombi but protected by CRO. H&E staining showed pathological changes in the intestinal tract of mice in the IRI group, as opposed to intestinal villi that were more intact in the CRO group (Figure 4E,F). These findings suggest that intestinal IRI‐induced microthrombi produced a hypoxic environment, and that this environment together with enterobacteria destroyed the intestinal barrier and hence increased serum LPS levels. CRO inhibited enterobacteria growth, and consequently, secured the intestinal barrier. Furthermore, ELISA testing demonstrated a decrease in both cf‐DNA and H3Cit levels in the CRO group (Figure 4G,H), consistent with the decreased levels of LPS seen in this group, indicating that CRO restrained LPS‐induced NETs. Moreover, CRO ameliorated the microcirculatory disturbances seen in the intestine and limbs of mice with IRI (Figure 4I–L). These data suggest that CRO can improve blood flow and alleviate IRI by reducing the release of NETs stimulated by intestinal LPS entry.

### 3.4 Inhibition of NETs Improved Intestinal Microcirculation Disorders

**FIGURE 5:**
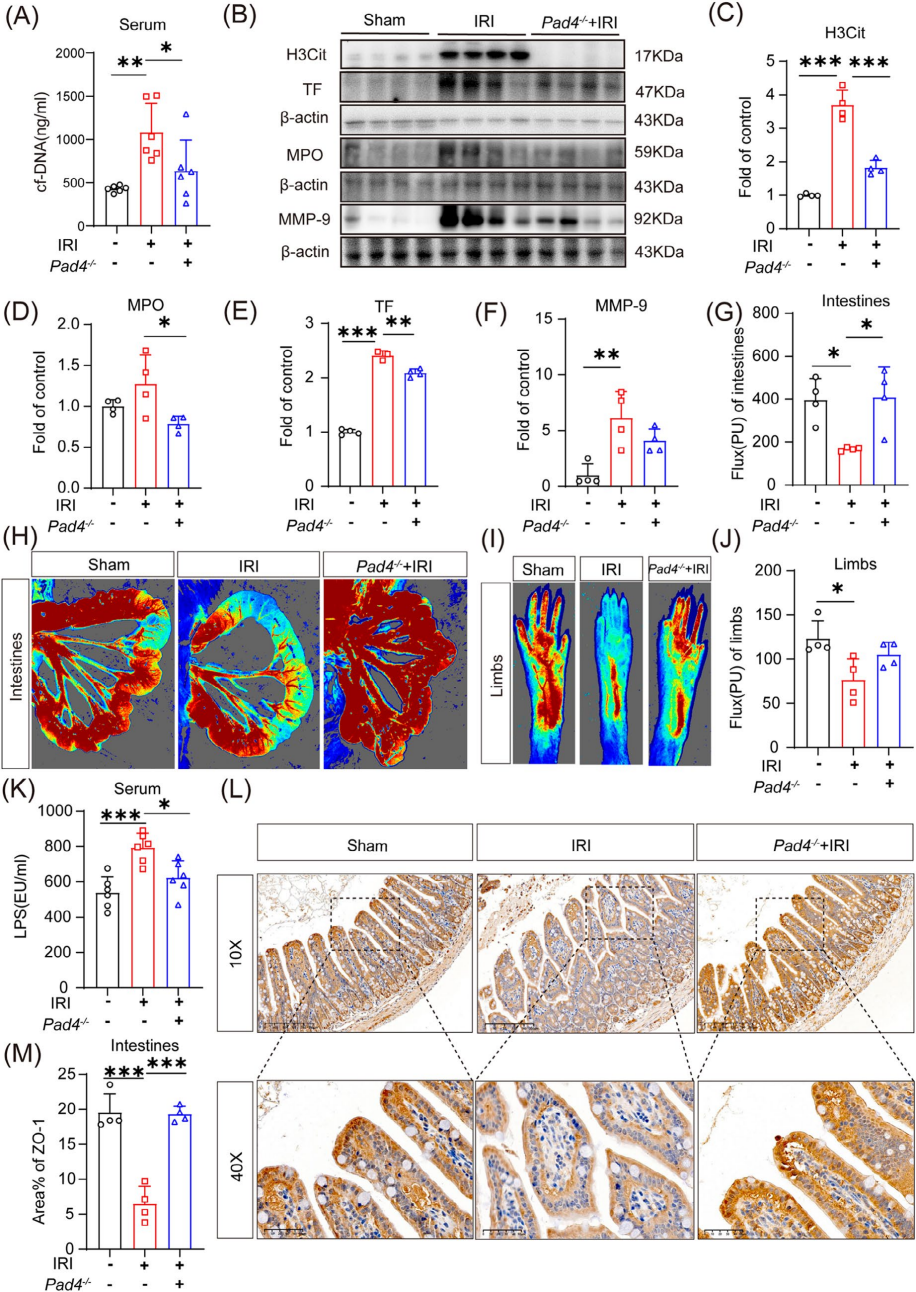
Peptidyl arginine deiminase 4 knockout (*Pad4* ^−/−^) improved intestinal microcirculation after intestinal ischemia reperfusion injury (IRI). (A) Detection of cell‐free (cf)‐DNA level in serum by ELISA (*n* = 6). (B–F) Detection of citrullinated histone H3 (H3Cit), myeloperoxidase (MPO), tissue factor (TF), and matrix metalloproteinase (MMP)‐9 expression in intestines by Western blot (*n* = 4). (G–J) Doppler flowmetry of intestinal and peripheral circulatory blood flow (*n* = 4). (K) Level of lipopolysaccharide (LPS) in serum detected using ELISA (*n* = 6). (L, M) Expression of intestinal tight junction protein zonula occludens (ZO)‐1 by immunohistochemistry (*n* = 4). Scale bar = 200 μm. *p < 0.05, **p < 0.01, and ***p < 0.001 versus sham group (one‐way ANOVA).

PAD4 is essential for the creation of NETs. Within 8 h after reperfusion, *Pad4* ^*−/−*^ mice demonstrated lower serum cf‐DNA levels (Figure 5A) and lower intestinal H3Cit, MPO, and TF levels (Figure 5B–E) than WT mice. In addition, MMP9 levels were significantly elevated in the intestines of WT mice but not in the intestine of *Pad4* ^*−/−*^ mice (Figure 5F). Intestinal and lower limb blood flow was similar in *Pad4* ^*−/−*^ mice and WT mice (Figure 5G–J); however, serum LPS levels were lower in *Pad4* ^*−/−*^ mice than in WT mice (Figure 5K). Mechanistically, ZO‐1 levels were preserved in *Pad4* ^*−/−*^ mice (Figure 5L,M). Taken together, these results suggest that the remission of microcirculatory disturbances after IRI in *Pad4* ^*−/−*^ mice could protect the intestinal mucosal barrier, alleviating microcirculatory impairment.

### 3.5 SHp‐DNase1 Prevented Intestinal IRI‐Induced Microcirculation Disorders

**FIGURE 6:**
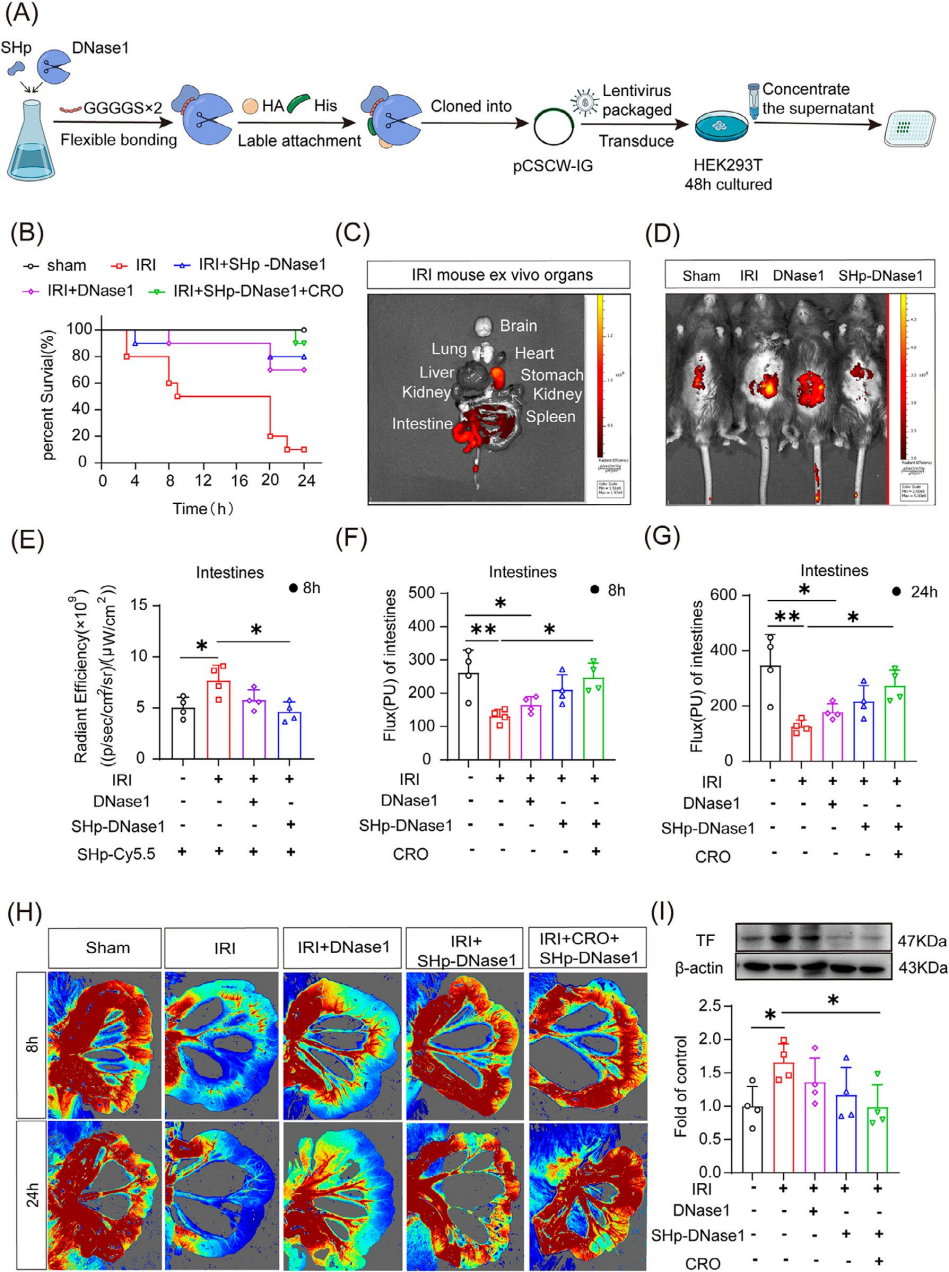
Stroke‐homing peptide (SHp)‐DNase1 therapy protected against intestinal ischemia reperfusion injury (IRI)‐induced microcirculatory dysfunction. (A) Preparation of SHp‐DNase1. (B) Survival rate within 24 h (*n* = 10, *p* < 0.0001 Sham versus IRI, *p* = 0.0053 IRI versus DNase1, *p* = 0.0025 IRI versus SHp‐DNase1, *p* < 0.0001 IRI versus SHp‐DNase1 + CRO). (C) Mice in the IRI group were injected with SHp‐Cy5.5 through the tail vein 2 h before imaging (at 6 h of reperfusion), and the organs were then isolated for observation via In Vivo Imaging System. (D, E) SHp‐Cy5.5 was injected into the tail vein and observed via in vivo imaging (*n* = 4). (F–H) Intestinal blood flow after 8 or 24 h of reperfusion, as observed by Doppler flowmetry (*n* = 4). (I) Western blotting of intestinal tissue factor (TF) expression (*n* = 4). *p < 0.05, **p < 0.01, and ***p < 0.001 versus sham group (one‐way ANOVA).

SHp is a peptide that targets the site of ischemia. To achieve targeted delivery of DNase1 to sites of ischemia caused by NETs, we designed the SHp‐DNase1 recombinant protein. The recombinant SHp‐DNase1 that we constructed (Figure 6A) significantly increased the survival rate in IRI mice within 24 h reperfusion, and only 1 death was seen in mice treated with SHp‐DNase1 plus CRO (Figure 6B). SHp‐Cy5.5 was found to accumulate only in the intestines and the stomach, not in other organs (Figure 6C). In vivo imaging showed that the fluorescence of SHp‐Cy5.5 was widely distributed in the abdomen of mice treated with DNase1 but was distributed only in a small part of the abdomen in mice treated with SHp‐DNase1 (Figure 6D,E). Intestinal blood flow caused by intestinal IRI was reversed by SHp‐DNase1 and by SHp‐DNase1 plus CRO (Figure 6F–H), and both SHp‐DNase1 and SHp‐DNase1 plus CRO reduced the expression of TF more effectively than DNase1 treatment alone (Figure 6I). Collectively, these findings suggest that SHp‐DNase1 and SHp‐DNase1 plus CRO were more effective than DNase1 alone. In addition, SHp‐DNase1 targeted only the site of ischemia and hypoxia and was not recruited to other organs, significantly improving the efficiency with which SHp‐carried DNase1 could perform its role. SHp‐DNase1 plus CRO inhibited NETs production by reducing LPS and also degraded the excessive release of NETs by targeting the ischemic site, alleviating intestinal IRI via a two‐pronged approach.

### 3.6 SHp‐DNase1 Was Required for the Clearance of LPS‐Induced NETs

**FIGURE 7:**
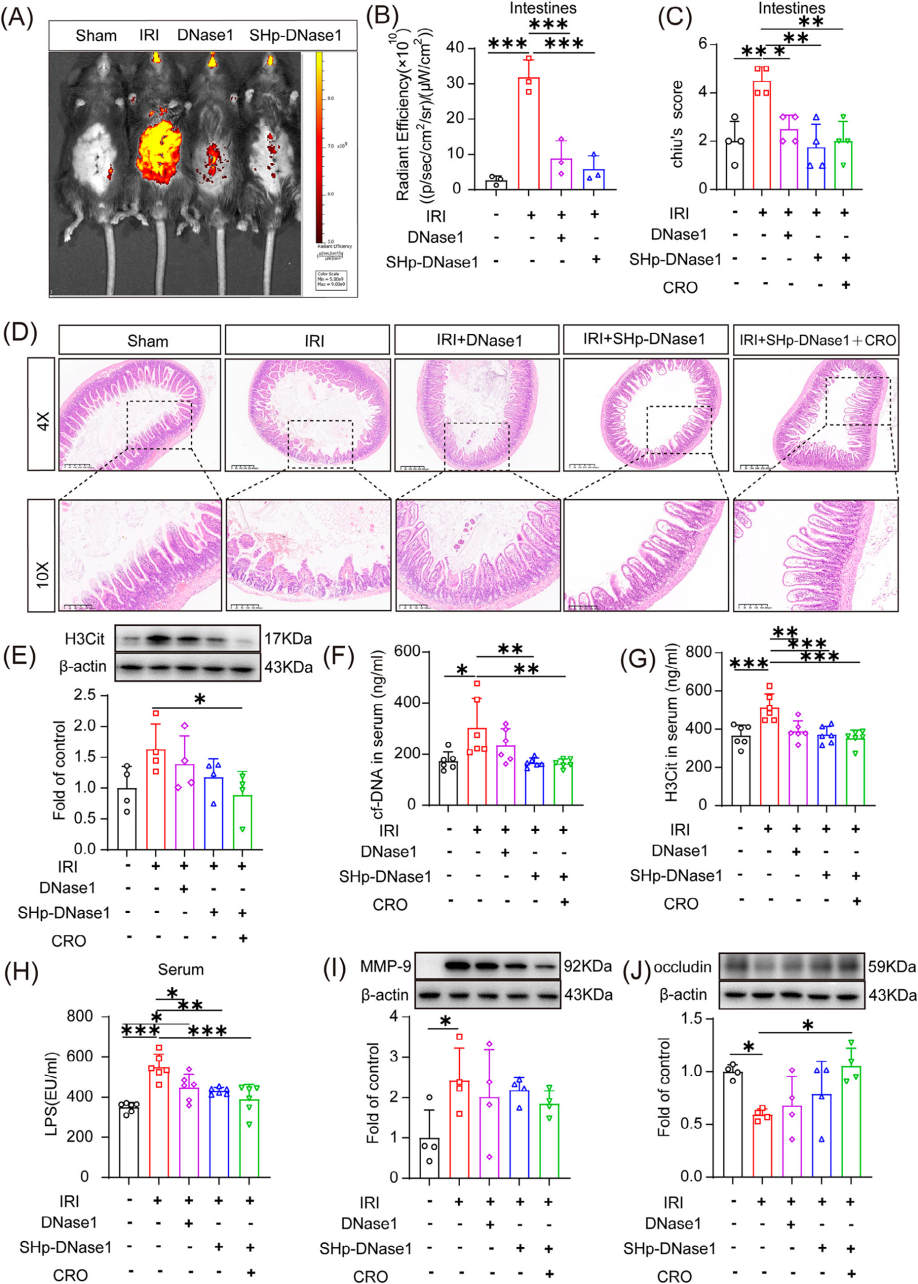
Stroke‐homing peptide (SHp)‐DNase1 reduced lipopolysaccharide (LPS)‐induced neutrophil extracellular trap formation after intestinal ischemia reperfusion injury (IRI). (A, B) Leakage of fluorescein isothiocyanate into the peritoneal cavity via the intestine was observed by In Vivo Imaging System at 8 h after reperfusion (*n* = 3). (C, D) H&E staining of intestines and corresponding Chiu's score (*n* = 4). 4× scale bar = 400 μm; 10× scale bar = 200 μm. (E) Detection of citrullinated histone H3 (H3Cit) levels in intestines by Western blotting (*n* = 4). (F, G) ELISA for the detection of cell‐free (cf)‐DNA and H3Cit at 24 h after reperfusion (*n* = 6). (H) Level of LPS in serum detected by ELISA (*n* = 6). (I, J) Expression of matrix metalloproteinase (MMP)‐9 and occludin in intestines by Western blotting (*n* = 4). *p < 0.05, **p < 0.01, and ***p < 0.001 versus sham group (one‐way ANOVA).

Live imaging by FITC gavage demonstrated that FITC leaked into the peritoneal cavity via the intestines, indicating that IRI led to intestinal barrier disruption. Both DNase1 and SHp‐DNase1 were able to attenuate the intestinal barrier damage caused by IRI, but SHp‐DNase1 was more effective than DNase1 (Figure 7A,B). Mice treated with SHp‐DNase1 or SHp‐DNase1 plus CRO had more intact intestinal villi and lower Chiu's scores compared with mice treated with DNase1 (Figure 7C,D). SHp‐DNase1 with or without CRO reduced the expression of cf‐DNA and H3Cit at 24 h after reperfusion (Figure 7E–G), and the same trends were seen for LPS and MMP‐9 levels (Figure 7H,I). We postulate that hypoxia and gut microbiota destroyed the intestinal barrier (Figure 7D,J), resulting in LPS leakage. This triggered NETs overproduction and subsequent inflammation, which damaged the intestine again. CRO was able to inhibit the gut microbiota and LPS leakage, and SHp‐DNase1 degraded NETs.

## 4 Discussion

In this study, we developed a DNase1‐targeted delivery system, SHp‐DNase1, to target the degradation of NETs, ameliorating intestinal microcirculatory disturbances in the context of intestinal IRI. We found that patients with intestinal IRI after CPB demonstrated a persistent elevation of NETs, suggesting that NETs are recruited by LPS from the damaged intestine, which in turn contributes to microthrombus formation and disruption of the intestinal mucosal barrier. Using a mouse model, we found that SHp‐DNase1 could target NETs more efficiently than DNase1 to alleviate intestinal microcirculatory disturbances and to decrease mortality. Moreover, we found that circulating LPS levels were reduced with the addition of CRO. Taken together, these results suggest that SHp‐DNase1 plus CRO may have strong therapeutic potential for the clinical treatment of intestinal IRI.

In patients undergoing cardiac surgery, CPB may result in decreased intestinal blood supply and oxygen delivery, increased intestinal permeability, and mucosal ischaemia [19], making gastrointestinal injury a common perioperative complication [29]. In this study, patients who underwent CPB and experienced intestinal IRI demonstrated high levels of NETs. In healthy patients, i‐FABP is located in the intestinal epithelial cells of the small intestinal villi. When the intestinal epithelium is damaged, I‐FABP is released into the circulation [19]. In this study, both I‐FABP and LPS serum levels were increased in patients with intestinal IRI. These findings suggest that intestinal barrier dysfunction is required for LPS leakage, which in turn contributes to NETs recruitment.

Several studies have assessed the effect of NETs in various disease models. It is found that NETs appeared to have a protective effect in a sepsis model induced by cecal ligation and puncture; however, NETs demonstrated adverse effects in the setting of sepsis combined with ischemia–reperfusion [30]. Mechanistically, removal of NETs was found to inhibit the adhesion, activation, and aggregation of platelets, precursors of thrombosis [31]. When thrombosis is seriously harmful or bacterial antigens (such as LPS) rather than bacteria cause pathological changes, the role of NETs in pathogen capture may be less important. Hence, knocking out PAD4 inhibits the formation of NETs, leading to less thrombosis and providing protective advantages [30], which is consistent with our current data.

Other studies have specifically assessed the relationship between NETs and intestinal IRI. In one study, NETs were found to facilitate intestinal microvascular endothelial ferroptosis by impairing Fundc1‐dependent mitophagy, which led to intestinal microcirculation disorder [12]. Another study found that NETs induced necrotising apoptosis of the intestinal epithelium through the TLR4/RIPK3/FUNDC1 pathway [32]. In the current study, we found that intestinal IRI stimulated the accumulation of NETs. This led to intestinal and peripheral microcirculation disorders and also destroyed the mucosal barrier, resulting in more LPS leaking into the blood and causing subsequent NETs accumulation.

To date, the use of DNase 1 to degrade NETs, thus limiting inflammation and microcirculation disturbance, has been considered a reasonable method for the supportive treatment of intestinal IRI [15, 16]. DNase1 does not decrease the release of NETs but instead degrades NETs into debris substrates by hydrolyzing the DNA skeleton, which can then be digested by macrophages [33]. However, DNase1 does not target NETs only in the intestine; it degrades extracellular DNA from any source [15]. Moreover, DNase1 also degrades ‘good’ NETs that catch bacteria and pathogens in healthy segments of the intestinal lumen. In this study, we therefore sought to develop a recombinant protein that could specifically target NETs in the ischemic intestine. We found that this SHp‐DNase 1 was more effective than DNase1 alone in improving intestinal microcirculation disorder and protecting the intestinal mucosal barrier. In addition, SHp‐DNase1 was found to significantly reduce mortality in mice with IRI, suggesting that this protein was successful in targeting NETs specifically in the ischemic intestine.

In addition to SHp‐DNase1, we also assessed the use of CRO in the mouse IRI model. CRO is a third‐generation cephalosporin that has strong antibacterial activity against both Gram‐negative and Gram‐positive bacteria [34]. CRO also has strong stability and a long half‐life (approximately 7 h) and is effective in treating a variety of infections, including intra‐abdominal infections in humans and animals [35, 36, 37]. LPS is the main and outermost component on the outer membrane of Gram‐negative bacteria [38], and research has shown that CRO can alleviate LPS‐induced sepsis [39]. CRO has also been shown to be effective in the treatment of brain IRI [40]. Additionally, in vitro studies have demonstrated that CRO can inhibit the formation of NETs to alleviate organ injury during sepsis [41]. In the current study, we found that CRO plus SHp‐DNase1 significantly reduced mortality, decreased the levels of serum and intestinal NETs, and improved microcirculation disturbances.

This study had several limitations. First, this study was based on the fact that SHp targets ischemia and hypoxia, but the specific mechanism of this agent requires further exploration. For example, in the previously described stroke model, SHp was found to be co‐located with some neurons but not with astrocytes [17]. In the IRI model, it is unknown which types of cells are specifically targeted by SHp. Second, the degradation of NETs depends on the comprehensive activities of two different DNases, namely DNase1 and DNase1‐like 3 (DNase1L3), which preferentially digest double‐stranded DNA and chromatin, respectively [42]. In future research, we plan to construct SHp‐DNase1L3 and SHp‐DNase1—DNase1L3 in an attempt to further degrade NETs.

## 5 Conclusion

In conclusion, our findings suggest that NETs are a core cause of reperfusion injury, microcirculation disturbance, and intestinal mucosal barrier damage after the restoration of intestinal blood flow. Based on this, we were able to successfully construct a recombinant protein, SHp‐DNase1, that specifically targets the sites of ischemia and hypoxia caused by NETs, thus alleviating intestinal microcirculatory disturbances and decreasing mortality. These results highlight the potential of using this agent in combination with an antibiotic such as CRO as a potential treatment for intestinal IRI.

## Author Contributions

Study design: Tian Tian, Wentao Liu, Lai Jin, and Zhongzhi Jia. Literature search: Tingting Liu, Xinrong Lv, Qingshan Xu, and Xiuting Qi. Data collection: Tingting Liu, Xinrong Lv, Qingshan Xu, Xiuting Qi, Shenghui Qiu, Na Shen, and Jing Cheng. Data analysis: Tingting Liu, Xinrong Lv, Xiuting Qi, Wentao Liu, Lai Jin, and Zhongzhi Jia. Data interpretation: Tingting Liu, Xinrong Lv, Xiuting Qi, Wentao Liu, Lai Jin, and Zhongzhi Jia. Figures: Tingting Liu, Xiuting Qi, Yaqi Luan, and Lan Jin. Drafting and revision: Tingting Liu, Xinrong Lv, Xiuting Qi, Wentao Liu, Lai Jin, and Zhongzhi Jia.

## Ethics Statement

All procedures were performed in accordance with regulations for the Care and Use of Laboratory Animals (The Ministry of Science and Technology of China, 2006). All animal experiments were approved by the Nanjing Medical University Animal Care and Use Committee (IACUC‐2305042) and were designed to minimise suffering and the number of animals used. All animal experiments were performed according to the ARRIVE (Animal Research: Reporting of In Vivo Experiments).

## Conflicts of Interest

The authors declare no conflicts of interest.

## Supporting information


**Table S1.** The characteristics of patients with cardiopulmonary bypass (CPB).

## Data Availability

The data that support the findings of this study are available from the corresponding author upon reasonable request.
