# Supplementary material for: Stroke‐Homing Peptide‐DNase1 Alleviates Intestinal Ischemia Reperfusion Injury by Selectively Degrading Neutrophil Extracellular Traps
Source: Cell Prolif. 2025 Feb 25;58(8):e70010. doi: 10.1111/cpr.70010 (PMC12336450; doi:10.1111/cpr.70010)
Supplement: Supplementary file 1 — Table S1. The characteristics of patients with cardiopulmonary bypass (CPB). [file CPR-58-e70010-s001.docx]

Supplemental Table S1. The characteristics of patients with cardiopulmonary bypass (CPB).

| Characteristic |  | Group(N=7) |
| --- | --- | --- |
| Age(years) | ≤50 | 2 |
|  | 51-60 | 1 |
|  | 61-70 | 1 |
|  | ≥71 | 3 |
| Sex | Male | 2 |
|  | Female | 5 |
| BMI (Kg/m^2^) | ＜18.5 | 0 |
|  | 18.5-23.9 | 4 |
|  | ＞23.9 | 3 |
| Duration CPB (minutes) | ＜100 | 0 |
|  | 大于100 | 7 |
